# Is There Variation in the Morphology of the Frontal Sinus in Individuals with Different Craniofacial Patterns? A Systematic Review with Meta-Analysis

**DOI:** 10.3390/dj12050143

**Published:** 2024-05-15

**Authors:** Erika Calvano Küchler, Maria Beatriz Carvalho Ribeiro de Oliveira, Isabela Ribeiro Madalena, Christian Kirschneck, Svenja Beisel-Memmert, Daniela Silva Barroso de Oliveira, Ângela Graciela Deliga Schroder, César Penazzo Lepri, Maria Angélica Hueb de Menezes-Oliveira, Guido Artemio Marañón-Vásquez

**Affiliations:** 1 Department of Orthodontics, University Hospital Bonn, Medical Faculty, 53111 Bonn, Germany; christian.kirschneck@uni-bonn.de (C.K.); svenja.memmert@ukbonn.de (S.B.-M.); 2 Department of Biomaterials, University of Uberaba, Uberaba 38010-200, MG, Brazil; mariabeatriz0406@edu.uniube.br (M.B.C.R.d.O.); isabela.madalena@uniptan.edu.br (I.R.M.); cesar.lepri@uniube.br (C.P.L.); angelica.hueb@uniube.br (M.A.H.d.M.-O.); 3 School of Dentistry, Presidente Tancredo de Almeida Neves University Center, São João del Rei 36307-251, MG, Brazil; 4 School of Dentistry, Federal University of Alfenas, Alfenas 37130-001, MG, Brazil; daniela.oliveira@unifal-mg.edu.br; 5 School of Dentistry, University of Tuiuti of Paraná, Curitiba 80230-901, PR, Brazil; dra.angela@digitalfaceradiologia.com.br; 6 Department of Pediatric Dentistry, School of Dentistry of Ribeirão Preto, University of São Paulo, Ribeirão Preto 77402-970, SP, Brazil; guido_amv@hotmail.com

**Keywords:** frontal sinus, malocclusion, maxilla, mandible

## Abstract

To evaluate differences in the morphology of the frontal sinus in adolescents and adults with different craniofacial patterns, searches up to April 2024 were conducted in six databases and other information sources to identify observational studies. Study selection, data extraction, and quality assessment using the NOS scale were performed independently by two reviewers. Random effects meta-analyses were conducted to estimate the difference in frontal sinus measurements between different craniofacial skeletal patterns (α = 0.05). The certainty of the evidence was evaluated according to GRADE. Fourteen studies were included in the review. All studies had methodological limitations that affected their quality. The syntheses showed that skeletal Class II subjects presented a significantly smaller width of the frontal sinus than skeletal Class I subjects (MD = 0.56; 95% CI: 0.38, 0.74; *p* < 0.0001; I^2^ = 3%). Skeletal Class III subjects showed a frontal sinus width (MD = −0.91; 95% CI: −1.35, −0.47; *p* < 0.0001; I^2^ = 36%) and area (MD = −28.13; 95% CI: −49.03, −7.23; *p* = 0.0084; I^2^ = 66%) significantly larger than those of the skeletal Class I subjects. The available evidence suggests a positive relationship between mandibular and frontal sinus size. There is limited evidence to make reliable estimates of the association of other craniofacial patterns and frontal sinus characteristics. These reported results are not conclusive and should be evaluated carefully due to the very low certainty of the evidence. The current evidence is scarce and consists of studies with methodological limitations; the results of the studies are often inconsistent, and the pooled estimates are imprecise. New high-quality research is still necessary.

## 1. Introduction

Humans have the following four pairs of paranasal sinuses: the maxillary, sphenoidal, ethmoidal, and frontal sinuses. The paranasal sinuses are mucosa-lined air spaces within the face and skull bones and grow in the same way as the bones [1]. The frontal sinuses are paired lobulated cavities, which are in the frontal bone posterior to the superciliary arches [2]. They are pneumatic cavities in the frontal bone and are one of the paranasal sinuses [3]. Each frontal sinus opens into the corresponding middle meatus via the infundibulum [2]; therefore, they are directly linked to the nasal cavity and located between the external and internal faces of the frontal bone [4,5,6]. The frontal sinus has a unique morphology. It is well known by its highly morphological variability, which is of interest in clinical surgery and forensic medicine. The examination of the frontal sinus shape is a valuable source to identify skeletal remains once it is a reliable method to predict the sex [5,7].

Skeletal malocclusions are a set of human craniofacial morphologic patterns which either exceed or exhibit a deficiency in the volume and proportion. The skeletal malocclusions result in an improper relationship of the jaws, changing the normal balance of the face. These changes may vary from minor to major deformities of skeletal origin [8]. The current literature has been showing that the frontal sinus development is associated to the maxilla and mandible development. Individuals with different skeletal malocclusions may have a different morphology of the frontal sinus [9,10,11,12,13,14,15,16,17,18,19,20,21,22,23]. 

Some studies investigated the association between sagittal skeletal malocclusions [12,13,14,16,17] and frontal sinus morphology, while other studies investigated its association with vertical skeletal malocclusions (vertical patterns of the face) [15]. Therefore, the present systematic review aimed to evaluate all the available literature that answers the following focused question: is there a difference in the morphology of the frontal sinus in adolescents and adults with different craniofacial patterns?

## 2. Materials and Methods

The protocol of the present systematic review was registered in the International prospective register of systematic reviews of the National Institute for Health Research, PROSPERO (CRD42023456902), available at https://www.crd.york.ac.uk/prospero/display_record.php?ID=CRD42023456902, accessed on 23 August 2023. This review was conducted and is reported in accordance with the Preferred Reporting Items for Systematic Reviews and Meta-Analyses (PRISMA) 2020 Statement [24].

### 2.1. Eligibility Criteria

Studies in accordance with the following PECOS strategy were included:

- Population (P): Adolescents and adults (over 12 years old). Non-clinical studies were not included.
- Exposure (E): Craniofacial patterns compatible with malocclusion (i.e., distobasal jaw relation, i.e., Class II relation, mesiobasal jaw relation, i.e., Class III relation, hyperdivergent vertical pattern, long face, etc.).
- Comparator (C): “Normal” craniofacial patterns (i.e., neutral basal sagittal relation, i.e., Class I relation, normodivergent vertical pattern, etc.).
- Outcome (O): Morphology of the frontal sinus, including two- and three-dimensional measurements (i.e., area, perimeter, width, height, shape, etc.).
- Study design (S): Observational studies. Baseline data from intervention studies were also considered of interest. Case reports, case series, reviews, letters to the editor, or expert opinion were not included.

It was pre-specified that studies would be grouped for synthesis according to reported craniofacial characteristics. Comparisons would be made between subjects “with craniofacial characteristics that deviate from normal” and subjects with “normal craniofacial characteristics”; for example, Class II individuals vs. Class I individuals, hyperdivergent individuals vs. normodivergent individuals, etc.

Additionally, and given that they answered the focused question of the review, it was decided to include studies that correlated craniofacial skeletal measurements and frontal sinus measurements.

### 2.2. Information Sources and Search Strategy

Searches were carried out until April 2024 in the following information sources: PubMed, Scopus, Web of Science, The Cochrane Library, Embase, LILACS, DANS EASY Archive (ex Opengrey), and Google Scholar. In this last source of information, the records were reviewed 100 by 100, until no potentially eligible studies were identified in addition to those already selected after searching the other sources. The reference lists of the selected studies were also reviewed. No experts on the matter were identified to consult for unpublished data or ongoing research.

Search strategies were constructed using controlled vocabulary (MeSH/Emtree terms) and free terms, which were combined using the Boolean operators “OR” and “AND”. Terms related to the concept’s frontal sinus and malocclusion/craniofacial skeletal alterations were selected. The search strategy was initially developed for PubMed and then adapted for the other sources of information according to the syntax rules of each of them. No language or publication date restrictions were established in the searches. Alerts were programmed into the databases to keep the searches updated. The search strategies used are presented in Supplementary Table S1.

### 2.3. Study Selection and Data Extraction

Identified studies were exported to the EndNote reference manager (https://web.endnote.com, accessed on 1 November 2022), where duplicates were automatically removed. Subsequently, the records were exported to the Rayyan software 1.3.3 (https://www.rayyan.ai, accessed on 1 November 2022), where the removal of duplicates was complemented manually. The selection process was also carried out in this software. Initially, two reviewers independently reviewed titles and abstracts to identify potentially eligible records. Then, the full texts were reviewed to define the selection. The reviewers held a consensus meeting to make a final decision, with the participation of a third reviewer in case of disagreements.

Once the studies were selected, the following information was extracted and tabulated: author and year of publication; age and ethnicity of the sample; imaging examination used for evaluations; craniofacial and frontal sinus measurements studied; sample size; results and conclusions of the authors. It was pre-established that, if essential data were not reported, the corresponding authors of the manuscripts would be contacted by email (5 attempts, 1 per week) to obtain the necessary information.

### 2.4. Assessment of Study Quality (Risk of Bias)

The quality of the included studies was evaluated according to the Newcastle–Ottawa Scale (NOS) [25]. The NOS is a scoring system that, for cohort and case-control studies, assigns 0–9 stars, divided into the following three aspects: selection of participants (4 stars), comparability (2 stars), and outcome (3 stars). In the case of cross-sectional studies, an adapted version of the NOS assigns 0–10 stars [26], with the following division: selection of participants (5 stars), comparability (2 stars), and outcome (3 stars).

The evaluations were carried out independently by two evaluators. Disagreements between the reviewers were resolved in a consensus meeting, with the participation of a third reviewer if disagreement persisted.

### 2.5. Methods for Synthesis and Evaluation of the Certainty of Evidence

Considering that frontal sinus measurements are reported as continuous data, the mean difference and its corresponding 95% confidence interval were pre-established as an effect measure for comparison between groups.

The data from the included studies were organized in tables to evaluate clinical/methodological homogeneity between them. The quantitative synthesis was carried out for methodologically homogeneous studies. Meta-analyses were performed using Jamovi 2.3 software to determine the mean difference for different frontal sinus measurements between subjects “with craniofacial characteristics that deviate from normal” and subjects with “normal craniofacial characteristics.” The random effects model was fitted to the data. The amount of heterogeneity was estimated using the DerSimonian–Laird estimator. The tau^2^ estimate, the Q-test for heterogeneity, and the I^2^ index were calculated as measures of heterogeneity. Sensitivity tests were performed to evaluate the robustness of the analyses. It was pre-established that subgroup analyses would be performed to explore possible sources of statistical heterogeneity, if possible and necessary. It was also considered to apply the rank correlation test and the regression test, which use the standard error of the observed results as a predictor, to check for asymmetries in the funnel plot in cases of meta-analysis including more than 10 studies. All estimates were conducted adopting a significance level of 5%.

Certainty of evidence was assessed for quantitatively synthesized results using Grading of Recommendations, Assessment, Development and Evaluation Pro software 1.3.3 (GRADEpro Guideline Development Tool, available online at gradepro.org, 14 November 2022). The risk of bias, inconsistency, indirectness, imprecision, and suspicion of publication bias were the aspects considered to rate the overall certainty of the evidence [27].

## 3. Results

### 3.1. Selection and Characteristics of the Studies

**Figure 1:**
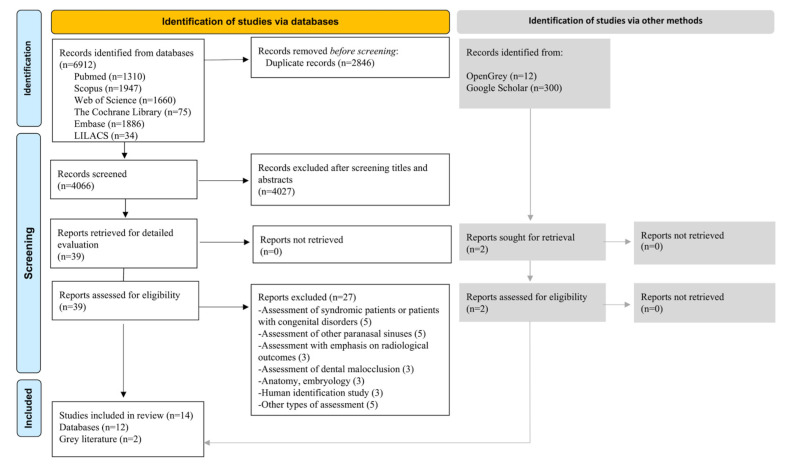
Flow diagram reporting items for systematic reviews.

The database search recovered a total of 6912 records. After removing duplicates, 4066 titles and abstracts were reviewed. Of a total of 39 records that were fully read, 27 were excluded (reasons provided in Figure 1 and a complete list of exclusions provided in Supplementary Table S2), leaving 12 records for inclusion. Of the searches via other methods, only two studies were evaluated for eligibility, and both were selected. Finally, 14 studies were included in the present review [9,10,11,12,13,14,15,16,17,18,19,20,21,22].

The studies were published between 2010 and 2024 and were conducted on adolescents and adults of different ethnicities. Nine of the fourteen studies performed evaluations of craniofacial and frontal sinus patterns on cephalometric radiographs [9,10,11,12,13,14,16,17,22], while the remainder performed their evaluations on conventional computed tomography or cone-beam computed tomography (CBCT) [15,18,19,20,21]. Two studies complemented their radiographic evaluations with postero-anterior radiographs [10,11].

Seven studies compared some measures of the frontal sinus according to skeletal malocclusion (determined by ANB angle values) [12,13,14,16,17,19,22], while only two compared them according to the vertical growth pattern [15,21]. Nine of the fourteen studies analyzed the correlation of craniofacial skeletal measurements with frontal sinus measurements [9,10,11,13,15,17,18,19,20].

### 3.2. Quality of Studies

All of the studies had methodological limitations that affected their quality. Two studies achieved a score of eight stars [18,22], five studies achieved a score of seven stars [10,11,14,17,19], one achieved four stars [12], and the rest achieved scores ranging between five and six stars [9,13,15,16,20,21]. The most affected item was the representativeness of the sample; no study carried out a random sampling process. The other two most affected items were the sample size and confounding control. Most studies did not report significant sample loss and used valid methods for the evaluation of outcomes and appropriate statistical methods. Detailed study quality assessment scores are presented in Table 1.

### 3.3. Results of Individual Studies and Synthesis

The characteristics, results, and conclusions of the individual studies are presented in Table 2. The results of the syntheses are presented below.

### 3.4. Relationship between Sagittal Craniofacial Patterns and Frontal Sinus Morphology

Meta-analyses were conducted to compare measurements of height, width, area, and frontal sinus index (height: width), evaluated on lateral cephalometric radiographs, between Class I vs. Class II and Class I vs. Class III skeletal malocclusions.

**Figure 2:**
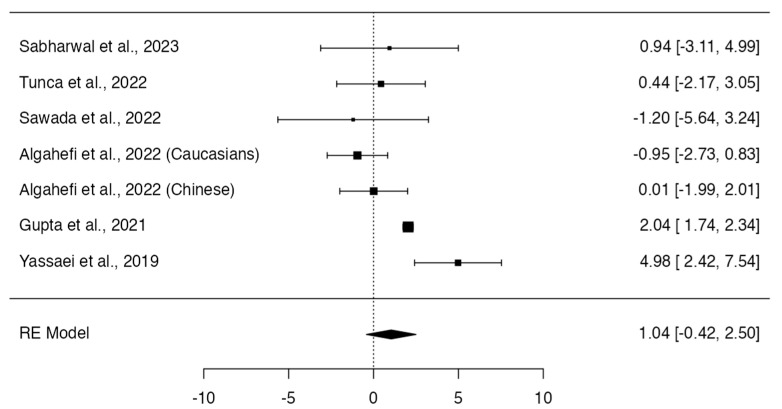
Meta-analyses performed to compare height measurements between Class I and Class II skeletal malocclusions [12,13,14,16,17,19,23].

**Figure 3:**
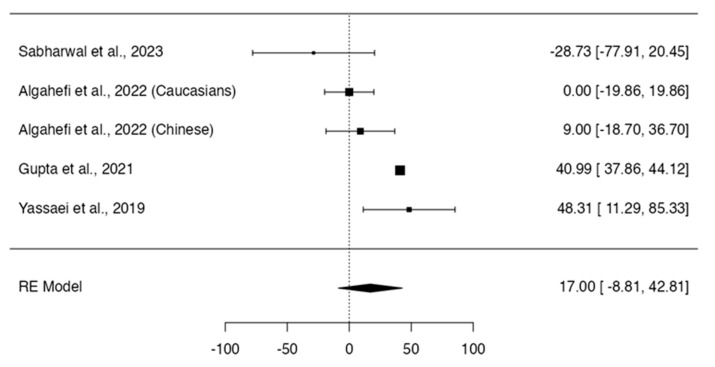
Meta-analyses performed to compare area measurements between Class I and Class II skeletal malocclusions [12,13,14,16,23].

**Figure 4:**
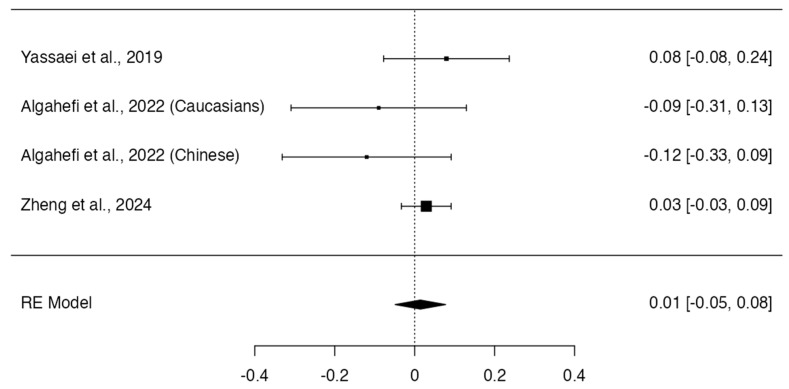
Meta-analyses performed to compare frontal sinus index measurements between Class I and Class II skeletal malocclusions [13,16,22,23].

**Figure 5:**
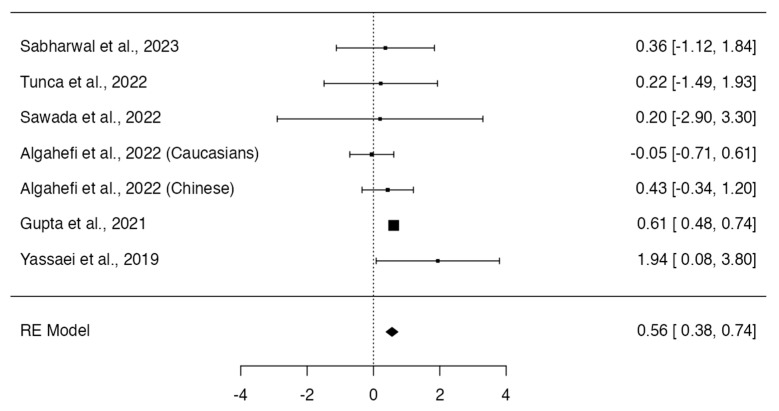
Meta-analyses performed to compare width measurements between Class I and Class II skeletal malocclusions [12,13,14,16,17,19,23].

Class II individuals did not show significant differences in height (*p* = 0.164; Q-test *p* = 0.0008; tau^2^ = 2.38; I^2^ = 74%; Figure 2) [12,13,14,16,17,19], area (*p* = 0.1966; Q-test *p* < 0.0001; tau^2^ = 656.38; I^2^ = 86%; Figure 3) [12,13,14,16], and frontal sinus index (*p* = 0.6589; Q-test *p* = 0.3509; tau^2^ = 0.00; I^2^ = 8%; Figure 4) [13,16,22] compared to Class I individuals. On the other hand, Class II subjects presented a significantly smaller width of the frontal sinus than Class I subjects (MD = 0.56; 95% CI: 0.38, 0.74; *p* < 0.0001; Q-test *p* = 0.4027; tau^2^ = 0.01; I^2^ = 3%; Figure 5) [12,13,14,16,17,22].

**Figure 6:**
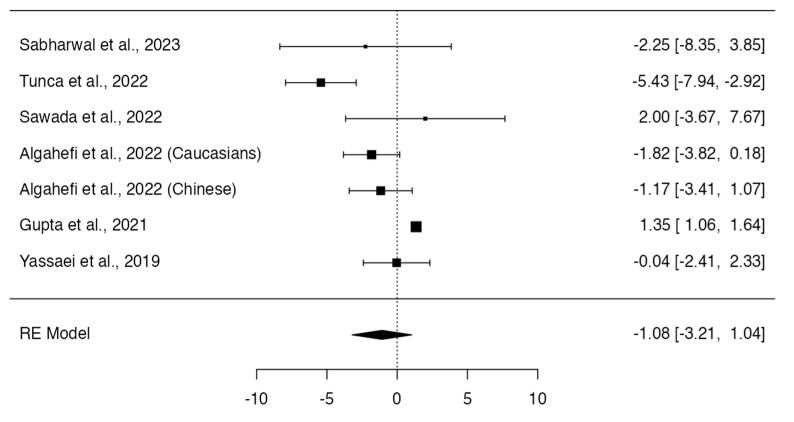
Meta-analyses performed to compare height measurements between Class I and Class III skeletal malocclusions [12,13,14,16,17,19,23].

**Figure 7:**
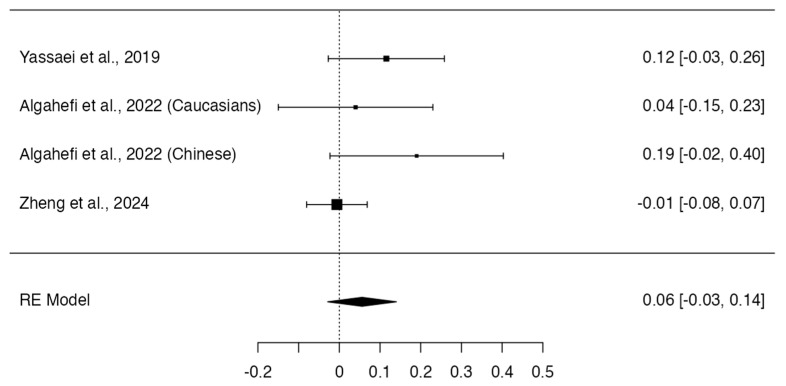
Meta-analyses performed to compare frontal sinus index measurements between Class I and Class III skeletal malocclusions [13,16,22,23].

**Figure 8:**
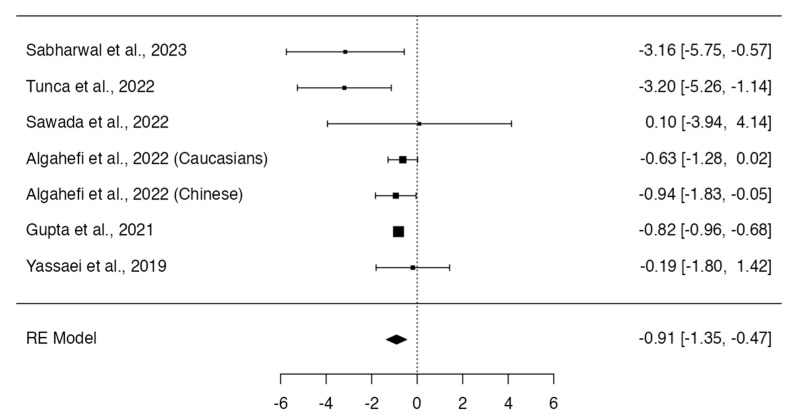
Meta-analyses performed to compare width measurements between Class I and Class III skeletal malocclusions [12,13,14,16,17,19,23].

**Figure 9:**
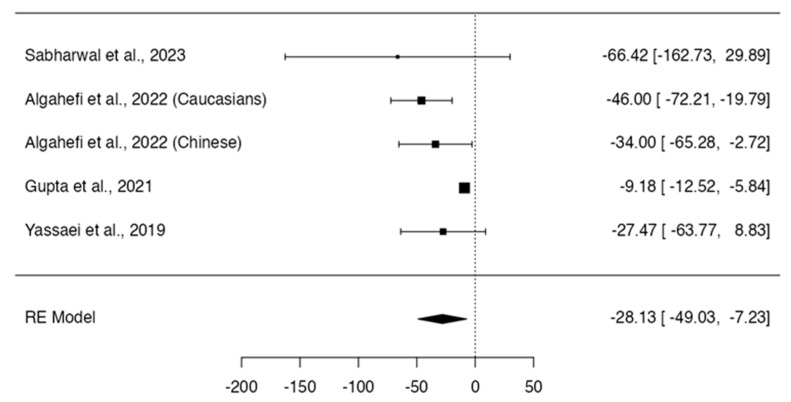
Meta-analyses performed to compare area measurements between Class I and Class III skeletal malocclusions [12,13,14,16,23].

Class III and Class I individuals did not show significant differences in the height (*p* = 0.318; Q-test *p* < 0.0001; tau^2^ = 5.95; I^2^ = 86%; Figure 6) [12,13,14,16,17,19] and frontal sinus index measurements (MD = 0.06; 95% CI: −0.03, 0.14; *p* = 0.1960; Q-test *p* = 0.2188; tau^2^ = 0.00; I^2^ = 32%; Figure 7) [13,16,22]. On the other hand, Class III subjects showed a width (MD = −0.91; 95% CI: −1.35, −0.47; *p* < 0.0001; Q-test *p* = 0.1508; tau^2^ = 0.11; I^2^ = 36%; Figure 8) [12,13,14,16,17,19] and frontal sinus area (MD = −28.13; 95% CI: −49.03, −7.23; *p* = 0.0084; Q-test *p* = 0.0179; tau^2^ = 316.08; I^2^ = 66%; Figure 9) [12,13,14,16] significantly larger than those of the Class I subjects.

The narrative synthesis showed that there is a low/moderate positive correlation of the sagittal measurements of the cranial base (i.e., S-N and AR-S) and mandible (i.e., Co-Gn and Go-Gn) with the height, width, area, and volume of the frontal sinus [13,17,18]. Sagittal measurements of the maxilla (i.e., Co-A and SNA) showed no correlation with sinus measurements [15,17,18], while sagittal measurements of the mandible (i.e., SNB) showed positive correlation with the sinus volume, total surface, and depth [18]. Corroborating these findings, and the estimates of the meta-analyses conducted, a moderate negative correlation has been reported between the measurement of the ANB angle and the height, width, and volume of the frontal sinus [17,18]. Additionally, an absence of correlation was reported between the sagittal measurements of the cranial base (i.e., S-N and AR-S), the mandibular body (i.e., Go-Gn), and the frontal sinus index [13]. Meta-analyses were not performed to calculate the pooled correlation due to methodological heterogeneity across the studies.

### 3.5. Relationship between Vertical Craniofacial Patterns and Frontal Sinus Morphology

No meta-analysis was conducted to compare groups according to the vertical skeletal pattern. Only one study [15], which performed its evaluations using CBCT, reported significant differences in the maximum anteroposterior distance according to the vertical pattern. The hypodivergent individuals showed significantly greater distances for this parameter than the normo- and hyperdivergent individuals. The maximum height and transverse length did not show differences according to the vertical pattern [15]. Similarly, frontal sinus volume was similar in subjects with a skeletal open bite and a deep bite [21].

The narrative synthesis evidenced a moderate negative correlation between the inclination of the skull base (i.e., SN/FH) and the height and width of the frontal sinus [11]. Some measures that evaluate the vertical facial skeletal component (i.e., SN/GoGn, SN/PP, and PP/MP) showed a moderate negative correlation with the width and/or area of the frontal sinus [10,15]. On the other hand, low/moderate positive correlation was reported between other of these measurements (i.e., gonial angle, SN/MP, N-Me, N-ANS, and ANS-Me) and the height, width, area, and/or volume of the sinus [10,13,15,18]. Measures that comprehensively evaluate the vertical component (i.e., the Jarabak Index and sum of the posterior angles) showed inconsistent results regarding their relationship with frontal sinus measurements [11,13,15].

Results on the correlation between mesiodistal width of the frontal sinus and craniofacial measurements were inconsistent [19,20].

### 3.6. Certainty of the Evidence

The certainty of the evidence was very low for all estimates (Table 3). The certainty of the evidence was decreased by one level for all evaluations, since the results used come from studies with methodological limitations that could compromise the validity of the results of the syntheses. The estimates for the outcome’s height, width (comparison between Class I vs. Class III), and area of the frontal sinus were affected for the inconsistency item. One or two levels of certainty were decreased due to lack of overlapping confidence intervals and/or high statistical heterogeneity. The syntheses of the outcomes of height, width, and index of the frontal sinus were also affected regarding the item indirectness, since individuals under 12 years of age were included in the analyses of some studies. Estimates for the outcomes of height, area (Class I vs. Class II), and frontal sinus index (Class I vs. Class III) were also affected by the item imprecision, since the 95% confidence intervals included zero (lack of effect), and additionally included values that demonstrate an important effect in one of the directions.

## 4. Discussion

Systematic reviews collect all possible studies related to the topic and review and analyze their results. During the process of the systematic review, the quality of the studies and their methodological limitations are assessed, and a statistical meta-analysis of the primary studies results is performed. A meta-analysis is a scientific method of combining and analyzing results from different studies [28]. The present systematic review and meta-analysis was performed aiming to investigate if there is a difference in the morphology of the frontal sinus between adolescents and adults with different craniofacial patterns. A previous systematic review investigated the association between sagittal skeletal malocclusions (skeletal Class I, II, and III) assessed in lateral cephalometric radiograph and frontal sinus morphology [18], while, in our systematic review, we performed a wider analysis and included more studies.

The frontal sinus can be featured and evaluated in different craniofacial images. The lateral cephalometric radiographs were the most used in the studies included here [9,10,11,12,13,14,16,17]. Posterior–anterior cephalogram was used in the studies by Tehranchi et al. [11] and Said et al. [10]. The study by Metin-Gürsoy et al. [15] was the only included study to perform the investigation using CBCT. CBCT is more accurate than conventional radiographies and is more appropriate for the assessment of craniofacial morphology.

Some authors proposed that the frontal sinus shape and measurements can be used as an additional predictor for forecasting skeletal malocclusion [29]. Skeletal Class I, II, and III are craniofacial sagittal patterns observed in humans. The prevalence of skeletal malocclusion Class II ranges from 2% to 63%, while the prevalence of skeletal malocclusion Class III ranges from 1% to 20%, depending of the studied population [30,31,32]. There is also some evidence in that frontal sinus morphology and size varies according to the ethnic groups [18,19,33].

The studies included here are from different populations. Sabharwal et al. [12] evaluated an Indian sample; Algahefi et al. [16] evaluated Yemeni and Chinese samples; Tunca et al. [17] and Metin-Gürsoy et al. [15] evaluated Turkish samples; Gupta et al. [14] evaluated a Nepalese sample; Yassaei et al. [13] and Tehranchi et al. [11] evaluated Iranian samples; Said et al. [10] evaluated North American samples; Serafim et al. [9] evaluated a Brazilian sample. The age of the samples of the primary studies also ranged, and it is known that the frontal sinus morphology and size also range according to the age, in which the growth peak of the frontal sinus occurs at approximately 1 year later the growth peak of the body [34]. It is important to mention that, although most studies evaluated samples of only adolescents and adults, some investigations evaluated broader age ranges, including participants who were probably before the peak of pubertal growth [9,19,20]. Because these samples also included individuals of interest, we chose to maintain these studies in the review. Some of these studies [19,20] were also included in the meta-analyses, since their results were not very distant from the others reported (it was verified, through sensitivity tests, that these studies were not the cause of the statistical heterogeneity observed) and because they increased the number of individuals in the analyses, which improved the precision of the estimates. In any case, since growing individuals were included (different from what was established in the eligibility criteria), the GRADE item indirectness was judged to be affected when evaluating the quality of the evidence.

In our meta-analysis, skeletal Class II individuals did not show significant differences in height, area, and frontal sinus index compared to skeletal Class I individuals. On the other hand, skeletal Class II individuals presented a significantly smaller width of the frontal sinus than skeletal Class I individuals. We also observed that skeletal Class III individuals showed a significantly larger width and frontal sinus area than skeletal Class I individuals. Furthermore, compared to Class I, Class III individuals did not show significant differences in height and frontal sinus index.

In our narrative synthesis analysis, we observed that sagittal measurements of the cranial base, such as S-N, AR-S, and mandible, such as Co-Gn and Go-Gn, are low/moderately positively correlated with the height, width, area, and volume of the frontal sinus [13,17,18]. Sagittal measurements of the maxilla, such as Co-A and SNA, showed no correlation with sinus measurements [15,17], while sagittal measurements of the mandible, such as SNB, showed positive correlation with sinus volume, total surface, and depth [18]. In agreement with the findings of the meta-analyses, a moderately negative correlation has been reported between the ANB angle measurement and the height, width, and volume of the frontal sinus [17,18]. Moreover, no correlation was reported between sagittal measurements of the cranial base, such as S-N and AR-S, and the mandibular body (Go-Gn) and the frontal sinus index [13]. It is important to emphasize that a limitation of our study is that meta-analyses were not performed to calculate the pooled correlation due to methodological heterogeneity across the included studies. A meta-analysis was also not conducted to compare groups according to vertical skeletal pattern, as only one study [15] evaluated the vertical patterns of the face using CBCT. The authors classified the patients as normodivergent, hyperdivergent, and hypodivergent. The authors observed that the anterior–posterior dimension of the frontal sinus decreases according to the vertical growth pattern, and they reported significant differences in the maximum anteroposterior distance according to the vertical pattern [15]. Similarly, frontal sinus volume was similar in subjects with a skeletal open bite and a deep bite [21].

The association observed here raises some interesting topics that should be discussed. The identification of some specific patterns could allow for studies that aim to identify characteristics involved in skeletal malocclusion prediction. The frontal sinus characteristics, together with some machine learning algorithms, could be used in orthodontic practice to predict maxilla and mandible growth and skeletal malocclusion establishment. The identification of these patterns could also have forensic and anthropological application once they improve the prediction of the face morphology based on the frontal sinus characteristics. For example, this knowledge can improve the recreation of a victim’s facial appearance from the frontal sinus morphology.

Although the present estimates suggest that there are differences between different craniofacial patterns for some frontal sinus dimensions, these results should be evaluated with caution due to the low quality of the evidence. Observational studies, by nature, provide low-quality evidence. Only in the case that the studies do not present significant threats to their validity is it possible to have greater confidence in the results reported by observational studies [27]. Most of the studies included in the present review had important methodological flaws, mainly related to lack of control for confounding, the possible non-representativeness of the sample, and the sample size. No study selected its sample randomly. It is difficult to make generalizations from the results of studies with convenience sampling due to the likelihood of selection bias [35]. It is also important to mention that, although the studies included in the meta-analyses were similar in terms of the groups compared and the methods of evaluation of the frontal sinus, some heterogeneity was observed in the characteristics of the samples. Although the subgroup analyses carried out did not demonstrate the contribution of the variables of ethnicity, sex, and age to the heterogeneity of the results, their influence cannot be ruled out. The results were inconsistent, making the evidence unreliable, probably due to the aforementioned factors [16,22]. Finally, it should be emphasized that the lack of association for some of the frontal sinus measures is also not entirely reliable due to the poor precision of the confidence intervals of the summary effects of the meta-analyses.

Briefly, the frontal sinus is of great interest for craniofacial growth, developmental biology, and orthodontic research. Future studies should attempt to use larger samples to investigate different craniofacial patterns besides sagittal malocclusions and investigate different populations such as European populations.

## 5. Conclusions

The available evidence suggests a positive relationship between mandibular size and frontal sinus size. Individuals with skeletal Class II malocclusions exhibit narrower frontal sinuses and those with skeletal Class III exhibit longer sinuses with greater area. There is limited evidence to make reliable estimates of the association of other craniofacial patterns and frontal sinus characteristics. In general, the results of the reported syntheses should be evaluated with caution due to the very low quality of the evidence. The current evidence is scarce, consisting of studies with methodological limitations; the results of the studies are often inconsistent, and the pooled estimates are imprecise. Due to the characteristics of the evidence, a response to the proposed focused review question is still inconclusive; therefore, new high-quality research is necessary. Future studies should consider evaluating powerful and population-representative samples and controlling for potential confounding variables.

## Figures and Tables

**Table 1:** Quality assessment.

Reference	Selection				Comparability	Outcome		Score
	Representativeness of the Sample	Sample Size Justified	Non-Respondents	Ascertainment of Exposure (max **)	Confounding Controlled (max **)	Outcome Assessment (max **)	Statistics	Total
Serafim et al. [9]			*	**		**	*	6/10
Said et al. [10]		*	*	**		**	*	7/10
Tehranchi et al. [11]			*	**	*	**	*	7/10
Sabharwal et al. [12]				*		**	*	4/10
Yassaei et al. [13]			*	*		**	*	5/10
Gupta et al. [14]		*	*	*	*	**	*	7/10
Metin-Gürsoy et al. [15]			*	*		**	*	5/10
Abate et al. [18]		*	*	**	*	**	*	8/10
Algahefi et al. [16]			*	**		**	*	6/10
Sawada et al. [19]		*	*	**		**	*	7/10
Tunca et al. [17]		*	*	**		**	*	7/10
Denny et al. [20]		*		*		**	*	5/10
Kumar and Pandian [21]				**		**	*	5/10
Zheng et al. [22]		*	*	**	*	**	*	8/10

**Table 2:** Characteristics of the included studies.

Author	Sample Age (Years) and Ethnicity	Image Exam	Exposure (Craniofacial Measurements)	Outcome(s) (Frontal Sinus Measurements)	Sample Size/Distribution	Authors’ Results and Conclusions
Serafim et al. [9]	8–14 (Brazilian women)	Lateral cephalogram	Co-Gn, Co-Go, Go-Gn, and Fg-Pg	Width Height	Total: 140	There was a low correlation between frontal sinus dimension and cephalometric measurements.
Said et al. [10]	Males older than 15 years; Females older than 13 years (North American)	Lateral cephalogram Posterior–anterior cephalogram	SN, SNA, SNB, ANB, Wits, MP-SN, PP-MP, ODI, U1-L1, Overbite, Overjet, U1-SN, U1-PP, and IMPA	Area	Total: 171 Class I (n = 20) Bimaxillary protrusion (n = 19) Open bite (n = 19) Class III with positive overjet (n = 19) Class III with anterior crossbite (n = 19) Class III with edge-to-edge (n = 5) Class II division 2 (n = 17) Class II division 1 with anterior contact (n = 19) Class II division 1 with no anterior contact (n = 19)	The measurements SN, MP-SN, and U1-PP were significantly associated with frontal sinus area.
Tehranchi et al. [11]	≥12 years (Iranian)	Lateral cephalogram Posterior–anterior cephalogram	SN-FH, Saddle, Articular, Gonial, Sum of posterior, Facial angle, Occ-SN, Pal-SN, Man-SN, SNA, SNB, ANB, Wits, Y-axis, and Jarabak index	Width Height Area	Total: 144	Greater dimensions of the frontal sinus were associated with decreased inclination of the anterior cranial base. There was also a correlation between frontal sinus dimensions and increased anterior facial height (sum of posterior angles, Pal-SN, and Jarabak index) in males and increased gonial angle in females.
Sabharwal et al. [12]	16–30 (Indian)	Lateral cephalogram	ANB was used to determine skeletal malocclusion Co-Gn (not related to sinus measurements)	Width Height Area	Total: 120 Class I (n = 18) Class II (n = 90) Class III (n = 12)	The width and area of frontal sinus are statistically significantly greater in Class III patients.
Yassaei et al. [13]	15–20 (Iranian)	Lateral cephalogram	ANB was used to determine skeletal malocclusion SN, AR-S, Go-Gn, SN-GoGn, Jarabak index, Sum of posterior angles, Gonial angle, Wits, and Occlusal plane	Width Height Area Ratio height/ width	Total: 116 Class I (n = 38) Class II with mandibular deficiency (n = 40) Class III with mandibular excess (n = 40)	Dimensions and surface area of the frontal sinus were greater in Class III patients. Dimensions and surface area of the frontal sinuses (except for the width) had a correlation with the mandibular body length, and the anterior and posterior cranial bases. Frontal sinus width had a correlation with the mandibular body length and the anterior cranial base.
Gupta et al. [14]	16–30 (Nepalese)	Lateral cephalogram	ANB was used to determine skeletal malocclusion Co-Gn (not related to sinus measurements)	Width Height Area	Total: 195 Class I (n = 65) Class II (n = 65) Class III (n = 65)	The frontal sinus area and width were statistically significant greater in Class III and smaller in Class II patients.
Metin-Gürsoy et al. [15]	17–38 (Turkish)	Cone-beam computed tomography (CBCT)	Jarabak’s ratio, SN/GoGn, N-Me, N-ANS, SNA, SN/PP, and Post. SUM	Height and width in the coronal plane; depth in the axial plane	Total: 87 Hypodivergent (n = 27) Normodivergent (n = 31) Hyperdivergent (n = 19)	The anterior–posterior dimension of the frontal sinus decreased according to the vertical growth pattern and was statistically correlated with vertical craniofacial structures.
Abate et al. [18]	12–40 (Italian)	Cone-beam computed tomography (CBCT)	SNA, SNB, ANB, SN, Total anterior height (AH), Upper AH, Lower AH, and ANS.PNS-Go.Me	Volume, total surface, linear maximum width, height, and depth	Total: 80	An increase in the depth, surface area, and volume of the frontal sinus was correlated with increasing SNB. The volume of the frontal sinus was increased in subjects with greater anterior skeletal dimension and with a greater SN. A decrease in ANB was correlated with an increase in frontal sinus volume.
Algahefi et al. [16]	Mean: 17.86 ± 3.49 (Caucasian); 17.32 ± 3.36 (Chinese) (Yemeni and Chinese)	Lateral cephalogram	ANB was used to determine skeletal malocclusion S-N, S-G, S-N/G-M, and Sg-N-G	Width Height Area Ratio height/width (index)	Total: 290 Caucasian: Class I (n = 65) Class II (n = 50) Class III (n = 30) Chinese: Class I (n = 65) Class II (n = 50) Class III (n = 30)	The frontal sinus area was statistically significant greater in Class III patients. The surface area and dimensions of the frontal sinus correlated with the S-N, S-G, S-N/G-M, and Sg-N-G.
Sawada et al. [19]	13.9 ± 1.3 (Japanese females)	Computed tomography	SNA, SNB, ANB, Facial angle, Y-axis, Gonial angle, FMA, PP-FH, SN, Wits, N-Me, Ar-Go, Ar-Me, and Go-Me	Breadth Height Depth Volume	Total: 53 Class I (n = 20) Class II (n = 20) Class III (n = 13)	No differences were observed in breadth, height, depth, or volume of the frontal sinus according to skeletal malocclusion.
Tunca et al. [17]	17–25 (Turkish)	Lateral cephalogram	ANB was used to determine skeletal malocclusion S-N, Co-A, and Co-Gn.	Width Height	Total: 60 Class I (n = 20) Class II (n = 20) Class III (n = 20)	The increase in frontal sinus height and width was correlated with the decrease in ANB and the increase in SN and Co-Gn. Frontal sinus dimensions are statistically significant greater in Class III patients.
Denny et al. [20]	20–54 (South Indians)	Cone-beam computed tomography (CBCT)	Cranial, nasal, maxillary, and mandibular width	Mesiodistal, anteroposterior, and superior–inferior measurements	Total: 142	Frontal sinus measurements correlated with nasal, cranial, maxillary, and mandibular width.
Kumar and Pandian [21]	20–35 (Indians)	Cone-beam computed tomography (CBCT)	Ar-Go, Ar-Go-Gn, and OP-HO	Volume	Total: 90 Normal overbite (n = 30) Skeletal open bite (n = 30) Skeletal deep bite (n = 30)	Frontal sinus volume was similar in subjects with a skeletal open bite and a deep bite.
Zheng et al. [22]	Males 17.15 ± 7.68; Females 18.35 ± 8.06 (Chinese)	Lateral cephalogram	ANB	Ratio heigth/width (index)	Total: 405 Class I (n = 204) Class II (n = 127) Class III (n = 74)	There was no statistically significant difference in bone classification, indicating that the frontal sinus depth had little relationship with dentoskeletal malocclusion.

**Table 3:** Assessment of the certainty of evidence.

							# of Participants	Absolute Effect	Overall Certainty
# of Datasets	Design of the Studies	Risk of Bias	Inconsistency	Indirectness	Imprecision	Other Considerations		MD (95% CI)	
Height (Class I vs. Class II)									
7	Observational	Serious ^a^	Very serious ^b,c^	Serious ^d^	Serious ^e^	None	606	MD: 1.04 (−0.42, 2.50)	⨁◯◯◯ VERY LOW
Width (Class I vs. Class II)									
7	Observational	Serious ^a^	Not serious	Serious ^d^	Not serious	None	606	MD: 0.56 (0.38, 0.74)	⨁◯◯◯ VERY LOW
Area (Class I vs. Class II)									
5	Observational	Serious ^a^	Very Serious ^b,c^	Not serious	Serious ^e^	None	546	MD: 10.0 (−8.8, 42.8)	⨁◯◯◯ VERY LOW
Frontal sinus index (Class I vs. Class II)									
4	Observational	Serious ^a^	Not serious	Serious ^d^	Not serious	None	639	MD: −0.01 (−0.05, 0.08)	⨁◯◯◯ VERY LOW
Height (Class I vs. Class III)									
7	Observational	Serious ^a^	Very serious ^b,c^	Serious ^d^	Serious ^e^	None	501	MD: −1.08 (−3.21, 1.04)	⨁◯◯◯ VERY LOW
Width (Class I vs. Class III)									
7	Observational	Serious ^a^	Serious ^b^	Serious ^d^	Not serious	None	501	MD: −0.91 (−1.35, −0.47)	⨁◯◯◯ VERY LOW
Area (Class I vs. Class III)									
5	Observational	Serious ^a^	Very serious ^b,c^	Not serious	Not serious	None	428	MD: −28.1 (−49.0, −7.23)	⨁◯◯◯ VERY LOW
Frontal sinus index (Class I vs. Class III)									
3	Observational	Serious ^a^	Not serious	Serious ^d^	Serious ^e^	None	544	MD: 0.06 (−0.03, 0.14)	⨁◯◯◯ VERY LOW

^a^ Data from some studies with methodological limitations that could have compromised the validity of the estimates. ^b^ Absence of overlapping confidence intervals. ^c^ I2 greater than 50%. ^d^ Individuals under 12 years of age were included in the analyses for some studies. ^e^ Confidence interval includes 0 (lack of effect), and additionally includes values that demonstrate an important effect in one of the directions (limit of the interval greater than 1 unit (mm, for distances), 0.1 (mm, for index), or 10 units (mm^2^, for area)).

## Data Availability

Not applicable.

## Supplementary Materials

The following supporting information can be downloaded at: https://www.mdpi.com/article/10.3390/dj12050143/s1, Table S1. Search strategies according to the information source; Table S2. Complete list of articles excluded after comprehensive reading and reasons for exclusion.
