# Supplementary material for: Is There Variation in the Morphology of the Frontal Sinus in Individuals with Different Craniofacial Patterns? A Systematic Review with Meta-Analysis
Source: Dent J (Basel). 2024 May 15;12(5):143. doi: 10.3390/dj12050143 (PMC11119789; doi:10.3390/dj12050143)
Supplement: Supplementary file 1 [file dentistry-12-00143-s001.zip › dentistry-2953812-supplementary.pdf]

Supplementary Table S1. Search strategies according to the information source.

Supplementary Table 1. Search strategies according to the information source.

Information source	Search strategy
PubMed	(Frontal Sinus[Mesh] OR Frontal Sinus*[Tiab]) AND (Malocclusion[Mesh] OR Malocclusion*[Tiab] OR Maxilla[Mesh] OR Maxill*[Tiab] OR Mandible[Mesh] OR Mandib*[Tiab] OR Dentofacial[Tiab] OR Dento-facial[Tiab] OR Dentoskeletal[Tiab] OR Dento-skeletal[Tiab] OR Craniofacial[Tiab])
Scopus	((INDEXTERMS({Frontal Sinus}) OR TITLE-ABS({Frontal Sinus} OR {Frontal Sinuses} OR {Frontal Air Sinus} OR {Frontal Air Sinuses})) AND (INDEXTERMS(Malocclusion OR Maxilla OR Mandible) OR TITLE-ABS(Malocclusion* OR Maxill* OR Mandib* OR Dentofacial OR Dento-facial OR Dentoskeletal OR Dento-skeletal OR Craniofacial)))
Web of Science	#1 TS=(Frontal Sinus*)<br>#2 TS=(Malocclusion* OR Maxill* OR Mandib* OR Dentofacial OR Dento-facial OR Dentoskeletal OR Dento-skeletal OR Craniofacial)<br>#3 #1 AND #2
The Cochrane Library	#1 MeSH descriptor: [Frontal Sinus] explode all trees<br>#2 (Frontal Sinus*):ti,ab,kw<br>#3 #1 OR #2<br>#4 MeSH descriptor: [Malocclusion] explode all trees<br>#5 MeSH descriptor: [Maxilla] explode all trees<br>#6 MeSH descriptor: [Mandible] explode all trees<br>#7 (Malocclusion* OR Maxill* OR Mandib* OR Dentofacial OR Dento-facial OR Dentoskeletal OR Dento-skeletal OR Craniofacial):ti,ab,kw<br>#8 #4 OR #5 OR #6 OR #7<br>#9 #3 AND #8
Embase	(('frontal sinus'/exp OR 'frontal sinus*':ab,ti) AND ('malocclusion'/exp OR 'maxilla'/exp OR 'mandible'/exp OR 'malocclusion*':ab,ti OR 'maxill*':ab,ti OR 'mandib*':ab,ti OR 'dentofacial':ab,ti OR 'dento-facial':ab,ti OR 'dentoskeletal':ab,ti OR 'dento-skeletal':ab,ti OR 'craniofacial':ab,ti))
LILACS	(mh:("Frontal Sinus") OR tw:("Frontal Sinus" OR "Frontal Sinuses" OR "Frontal Air Sinus" OR "Frontal Air Sinuses")) AND (mh:(malocclusion OR maxilla OR mandible) OR tw:(malocclusion* OR maxill* OR mandib* OR dentofacial OR dento-facial OR dentoskeletal OR dento-skeletal OR craniofacial)) AND (db:("LILACS"))
DANS EASY Archive / Google Scholar	-frontal sinus AND Malocclusion<br>-frontal sinus AND Maxilla<br>-frontal sinus AND Mandible

Supplementary Table S2. Complete list of articles excluded after comprehensive reading and reasons for exclusion

Title	Authors	Publication date	Reference	Reason for exclusion
Development of the frontal sinus after frontocranial remodeling for craniostenosis in children in a group of adolescent Caucasians and Chinese in diferent skeletal malocclusions: a cross-sectional cephalometric study	Arnaud, E.; Marchac, D.; Renier, D.	1994 Apr	Annales de Chirurgie Plastique Esthetique - Volume 39, Issue 2, pp. 151-161	A
Cone beam computer tomography for paranasal sinus imaging malocclusions: a cross-sectional cephalometric study	Eggers, G.;	2011 Jan	International Journal of Computer Assisted Radiology and Surgery - Volume 6, Issue 0, pp. S205-S206	C
Morphometric variants of the paranasal sinuses in a Mexican population: expected changes according to age and gender	Jasso-Ramirez, N. G.; Elizondo-Omaña, R. E.; Treviño-Gonzalez, J. L.; Quiroga-Garza, A.; Garza-Rico, I. A.; Aguilar-Morales, K.; Elizondo-Riojas, G.; Guzmán-Lopez, S.;	2022 Jan	Folia morphologica - Volume 0, Issue 0, pp	B
The Effect of Nasal Septal Deviation on Frontal and Maxillary Sinus Volumes and Development of Sinusitis	Karataş, D.; Koç, A.; Yüksel, F.; Doğan, M.; Bayram, A.; Cihan, M. C.;	2015 Jan	The Journal of craniofacial surgery - Volume 26, Issue 5, pp. 1508-1512	A
Morphological variation of the paranasal sinuses in strepsirrhines	Nishimura, T. D.; Lebrun, R.; De Leon, M. P.; Zollikofer, C. P. E.	2011 Jan	American Journal of Physical Anthropology - Volume 144, Issue 0, pp. 225-226	B
Comparison of the visibility of the anatomical structures of the facial skeleton in panoramic zonography and linear tomography	Paukku, P.; Totterman, S.; Hallikainen, D.;	1983 Jan	European Journal of Radiology - Volume 3, Issue 3, pp. 177-179	C

The relationship between anatomical variations of the sino-nasal region and chronic sinusitis extension in children	Al-Qudah, M.;	2008 Jun	Int J Pediatr Otorhinolaryngol - Volume 72, Issue 6, pp. 817-21	A
The Relationships Between Craniofacial Structure and Frontal Sinus Morphology: Evaluation With Conventional Anthropometry and CT-Based Volumetry	Aslıer, N. G. Y.; Zeybek, G.; Karabay, N.; Keskinoğlu, P.; Kiray, A.; Sütay, S.; Ecevit, M. C.;	2020 Dec	Ear Nose Throat J - Volume 99, Issue 10, pp. 637-647	A
Anatomy, Head and Neck, Nose Sinuses	Henson, B.; Drake, T. M.; Edens, M. A.;	2022 Jan	StatPearls - Volume 0, Issue 0, pp.	E
Computer-assisted anatomical evaluation of the nasal sinuses in 2-3 years old children	Liao, D. H.;	2017 Sep	Lin Chung Er Bi Yan Hou Tou Jing Wai Ke Za Zhi - Volume 31, Issue 17, pp. 1355-1358	B
Computer-assisted anatomical evaluation of the nasal sinuses in infants	Liao, D.; Duan, C.;	2011 Dec	Lin Chung Er Bi Yan Hou Tou Jing Wai Ke Za Zhi - Volume 25, Issue 23, pp. 1057-9	B
Comments on the frontal sinus and mandibular growth prediction	Meithke, R. R.;	1993 Jan	Am J Orthod Dentofacial Orthop - Volume 103, Issue 0, pp. 20a-22a	D
Prevalence of skeletal and dental anomalies and normal variants seen in cephalometric and other radiographs of orthodontic patients	Tetradis, S.; Kantor, M. L.;	1999 Nov	Am J Orthod Dentofacial Orthop - Volume 116, Issue 5, pp. 572-7	D
Considerations on variation of size of frontal sinuses	Walander, A.;	1965 Jul	Acta Otolaryngol - Volume 60, Issue 0, pp. 15-22	E
The Age-Related Development of Maxillary Sinus in Children	Degermenci, M.; Ertekin, T.; Ulger, H.; Acer, N.; Coskun, A.;	2016 Jan	Journal of Craniofacial Surgery - Volume 27, Issue 1, pp. E38-E44	B
Lack of significant volumetric alteration after rapid maxillary expansion supports the use of frontal sinuses for human identification purposes	Furtado, G. C.; Pompeo, D. D.; Furtado, A.; Paranhos, L. R.; Franco, A.; Lima-Rivera, L. M.;	2018 Mar	Journal of Forensic Radiology and Imaging - Volume 12, Issue 0, pp. 64-67	F

Morphometric evaluation of the frontal sinus in relation to age	Fatu, C.; Puisoru, M.; Rotaru, M.; Truta, A. M.;	2006 May	Annals of Anatomy-Anatomischer Anzeiger - Volume 188, Issue 3, pp. 275-280	G
A Prospective Study to Determine and Compare the Sizes of the Frontal Sinus by Age and Gender	Gurjar, A. S.; Gurjar, M.;	2022 Jan	Advances in Human Biology - Volume 12, Issue 1, pp. 38-41	F
Broadening the Scope and Utility of Frontal Sinus Morphology for Predicting the Growth Pattern and Skeletal Malocclusion in Cleft Lip and Palate Cases	Jadhav, V. V.; Daigavane, P.; Kamble, R.; Shrivastav, S.; Tiwari, M.;	2021 Jan	Journal of Pharmaceutical Research International - Volume 33, Issue 51, pp. 173-179	A
Dental and facial skeletal characteristics and growth of males and females with Class II, Division 1 malocclusion between the ages of 10 and 14 (revisited) - Part I: Characteristics of size, form, and position	Rothstein, T.; Yoon-Tarlie, C.;	2003 Mar	American Journal of Orthodontics and Dentofacial Orthopedics - Volume 117, Issue 3, pp. 320-332	D
Pharyngeal airway space and frontal and sphenoid sinus changes after maxillomandibular advancement with counterclockwise rotation for class II anterior open bite malocclusions	Prado, F. B.; Rossi, A. C.; Freire, A. R.; Groppo, F. C.; De Moraes, M.; Caria, P. H. F.;	2012 Feb	Dentomaxillofacial Radiology - Volume 41, Issue 2, pp. 103-109	G
Ethnic Variation of Sinonasal Anatomy on CT Scan and Volumetric Analysis	Mokhasanavisu, V. J. P.; Singh, R.; Balakrishnan, R.; Kadavigere, R.;	2019 Nov	Indian Journal of Otolaryngology and Head & Neck Surgery - Volume 71, Issue 0, pp. 2157-2164	E
Effect of Vertical Growth Pattern on Maxillary and Frontal Sinus Sizes	Goymen, M.; Buyuknacar, G. B.; Gulec, A.;	2019 Sep	European Journal of Therapeutics - Volume 25, Issue 3, pp. 197-200	G
A study of the characteristics of mandibular growth in relation to the size of frontal sinus	Lee, S. Y.; Kim, S. C.;	1995 Mar	Journal of Dental Research - Volume 74, Issue 3, pp. 990-990	G
Anatomical variations of maxillary sinus: a cone-beam computed tomography study	Yazdani, J.; Parnia, F.; Torab, A.;	2018 Jun	Biointerface Research in Applied Chemistry - Volume 8, Issue 3, pp. 3298-3301	C

A CBCT based analysis of the correlation between volumetric morphology of the frontal sinuses and the facial growth pattern in caucasian subjects. A cross-sectional study	Abate, A.; Gaffuri, F.; Lanteri, V.; Fama, A.; Ugolini, A.; Mannina, L.; Maspero, C.;	2022 Feb	Head & Face Medicine - Volume 18, Issue 1, pp.	F
Evaluation of Dimensional Changes in Maxillary and Frontal Sinus in Adult PatientsWith Anterior Open Bite and Normal Overbite: A Retrospective Cone Beam Computed Tomography (CBCT) Study.	Sivasankari R S S, Nayeemullah Khan, Ratna Parameswaran, Srinivasan Boovaraghavan, Manini Nagi	2024 Feb	Cureus. 2024 Feb 6;16(2):e53710.	G

A - Assessment of syndromic patients or patients with congenital disorders
B - Assessment of other paranasal sinuses
C - Assessment with emphasis on radiological outcomes
D - Assessment of dental malocclusion
E - Anatomy, embryology
F - Human identification study
G - Other types of assessment
